# Modeling magnetic properties of cobalt nanofilms used as a component of spin hybrid superconductor–ferromagnetic structures

**DOI:** 10.3762/bjnano.16.110

**Published:** 2025-09-08

**Authors:** Aleksey Fedotov, Olesya Severyukhina, Anastasia Salomatina, Anatolie Sidorenko

**Affiliations:** 1 Modeling Structures and Functional Materials Group, Institute of Mechanics, Udmurt Federal Research Center, Ural Division, Russian Academy of Sciences, Baramzinoy 34, Izhevsk 426067, Russia; 2 Nanotechnology and Microsystems Engineering Department, Kalashnikov Izhevsk State Technical University, Studencheskaya 7, Izhevsk 426069, Russia; 3 Institute of Electronic Engineering and Nanotechnologies, Technical University of Moldova, 3/3 Academiei St., Chisinau, 2028, Moldova; 4 Moscow Institute of Physics and Technology, 9, Institutskiy per., Dolgoprudny, 141701, Russia

**Keywords:** ferromagnetic properties, LAMMPS, mathematical modeling, MEAM, molecular dynamics, spin dynamics

## Abstract

The paper presents a mathematical model for studying the magnetic behavior of atoms, which takes into account spin and interatomic interactions. Two problems were solved by means of mathematical modeling. At the first stage, the problem of modeling a small nanoscale system (500 atoms) consisting of cobalt atoms was solved. The purpose of this stage of computational experiment was to check the convergence of the solution and compare the obtained data with the results of other studies. The performed calculations and satisfactory correspondence to the previously obtained data confirmed the adequacy of the applied mathematical model. The second stage of numerical studies was devoted to the analysis of the magnetic behavior of cobalt nanofilms of different thicknesses. It was shown that the film thickness has a significant influence on the magnetic parameters of the modeled nanoscale systems. It was found that the magnetic energy and magnetization norm of the system change in a nonlinear manner with increasing number of crystalline layers of the nanofilm. The peaks found on the graph of the magnetization rate change can be caused by surface effects in thin films and the formation of Neel domain walls.

## Introduction

Thin film structures [1–2] are increasingly employed each year in a wide range of applications, serving as functional [3–4], reinforcing, light-reflecting, conductive, and dielectric materials. Their utilization extends to contacts, printed circuit boards, and integrated circuit elements in microelectronics, as well as to the fabrication of optical filters, the component base of optoelectronics, and advanced lithographic processes. Due to active experimental and theoretical research on thin films, significant progress has been made in recent years. Since the information in the field of thin film technologies is updated quite rapidly, there is a need for a thorough study and optimization of the main technological processes that are currently used, as well as fundamental features of the thin film formation processes with new composition and coatings of various types. These types of nanomaterials are very promising (in terms of computational performance and energy dissipation efficiency) for use in superconducting digital technologies [5–7] based on Josephson junctions.

It is well established that the properties of nanostructures can significantly differ from those of bulk samples. Currently, close attention is paid to thin-film magnetic structures, which include cobalt and iron [8–10]. Thus, in [1], the crystal structure and composition of Co–Ni–Fe films were evaluated, and it was found out how the deposition rate affects the conversion of a weak magnetic field into magnetic induction. In addition, thin-film structures based on Fe and Co are among the most promising materials that can be applied in the creation of magnetic heads for recording and reading information, memory cells, and other devices [11] which utilize magnetic properties of materials. Magnetic properties of nanofilms [12–13], in particular cobalt nanofilms, represent an important subject of research in both theoretical and practical fields of materials science. These properties depend not only on the composition, but also on factors such as film thickness, which in turn affects their application in microelectronics, spintronics, and other high-tech fields.

The aim of this article is to model the magnetic properties of cobalt nanofilms of different thicknesses and to reveal the main interdependence mechanisms of dimensional, structural, and magnetic subsystems. The proposed modeling methodology and the conducted studies make it possible to analyze the regularities determining the magnetic properties of thin films, which will further make it possible to optimize them for specific applications and tasks. The present work is a development of earlier publications by the authors [14–16].

The cobalt thin films studied in this work can be a component of superconductor–ferromagnetic hybrid nanostructures, which are the basis for the formation of Josephson contacts [13]. These nanomaterials are widely used [17–19] in the creation of individual qubits and quantum computers in general, superconducting microcircuits and interferometers, single photon detectors [20] and other devices of quantum electronics and spintronics.

## Mathematical Model for Studying the Magnetic Behavior of Atoms

To conduct computational experiments, we used a mathematical model describing the coordinated motion of atoms and the change of their spin vectors. The spin vector of an atom in this case was understood as the intrinsic magnetic moment associated with the momentum of the atom, which was calculated as a vector sum of the spins of individual electrons included in its structure and their orbital moments.

The mathematical model of atomic displacement and changes in their magnetic moments is based on the Langevin [21] and Landau–Lifshitz–Hilbert [22–23] equations:


[1]

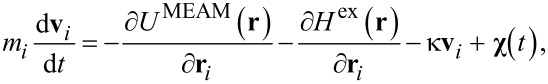




[2] $$\frac{\text{d}s_{i}}{\text{d}t}=\frac{1}{\left(1+\lambda^{2}\right)}\left(\left(\omega_{i}+\eta \left(t\right)\right)\times s_{i}+\lambda s_{i}\times \left(\omega_{i}\times s_{i}\right)\right),\;i=1,2,...,N$$

where *U*^MEAM^(**r**) is the force potential, the modified embedded atom method (MEAM) potential was used in this work; *H*^ex^(**r**) is the exchange interaction energy of spins; **r** = {**r**_1_,**r**_2_,…,**r***_N_*} is the generalized variable showing the dependence on the whole set of radial vectors of atoms; κ,λ are viscous friction force parameter and damping spin coefficient, respectively; **χ**(*t*), **η**(*t*) are white noise present in the description of atom motion processes and the behavior of their spins, respectively; and **ω***_i_* is the multiplication value of the gyromagnetic ratio and the local magnetic field [22].

It has been known for quite a long time [24–25] that the fluctuations of thermal and magnetic energy of an atomistic system can be described in the framework of the Langevin theory. To solve stochastic differential equations, which are the basis of this theory, random forces **χ**(*t*) and **η**(*t*), characterized by the following properties, are used:


[3] $$\langle \chi \left(t\right)\rangle =0,\langle \chi_{\alpha }\left(t\right)\chi_{\beta }\left(t^{\prime }\right)\rangle =\frac{2k_{\text{B}}T_{l}}{B}\delta_{\alpha \beta }\delta \left(t-t^{\prime }\right),$$

[4]

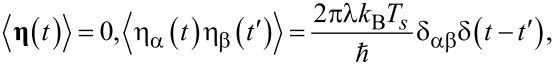



where *t* and *t*´ are different time points; α and β are components of the random force vector, for the three-dimensional case {α,β} = {*x*,*y*,*z*}; δ(*t* − *t*´) is the Dirac delta measure; *k*_B_ is the Boltzmann constant; 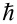 is the reduced Planck constant; *B* is the mobility value of the Brownian particle; and *T_l_* and *T_s_* are values of the thermodynamic and spin temperatures, respectively [26–27].

The potential of the modified embedded atom method [28–29] was based on the electron density functional theory. The magnitude of the potential *U*^MEAM^ depends on the set of atomic positions, which makes the potential multi-body model:


[5] $$U^{\text{MEAM}}\left(r\right)=\sum_{i}\left(F_{i}\left({\bar{\rho }}_{i}\right)+\frac{1}{2}\sum_{j,j\neq i}\phi_{ij}\left(r_{ij}\right)\right),\;i=1,2,...,N,$$

where *F_i_* is the immersion function of each atom in the electron gas created by electrons of all other atoms of the system; ϕ*_ij_*(*r_ij_*) is the pair potential function; *r_ij_* = |**r***_ij_*| = |**r***_j_* − **r***_j_*| is the distance between two atoms with numbers *_i_* and *_j_*; and 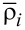 is the background electron density. When describing the electron density, different types of electron clouds of the atoms under consideration are taken into account, and a sufficiently large set of potential parameters is involved, which makes MEAM sufficiently accurate and allows it to be used in solving a wide range of atomistic modeling problems.

The exchange interaction energy of spins is used in mathematical models to describe the magnetic behavior of systems and allows us to reproduce their ferromagnetic and antiferromagnetic ordering. The total energy value is calculated according to the following expression:


[6] $$H^{\text{ex}}\left(r\right)=-\sum_{j,i\neq j}^{N}J\left(r_{ij}\right)s_{i}\cdot s_{j},$$

where *J*(*r_ij_*) is the exchange integral, the sign of which determines the type of interaction (ferromagnetic or antiferromagnetic); and **s***_i_* and **s***_j_* are spin vectors of individual atoms. Equation 6 provides a connection between the spatial and spin degrees of freedom of the system through the exchange integral in the form of the Bethe–Slater curve:


[7] $$J\left(r_{ij}\right)=4\varepsilon {\left(\frac{r_{ij}}{\delta }\right)}^{2}\left(1-\gamma {\left(\frac{r_{ij}}{\delta }\right)}^{2}\right)e^{-{\left(\frac{r_{ij}}{\delta }\right)}^{2}}\Theta \left(R_{c}-r_{ij}\right),$$

where ε,δ,γ are parametric coefficients of the model; Θ(*R_c_* − *r_ij_*) is the piecewise constant of the Heaviside function; and *R_c_* is the distance at which the exchange integral is clipped.

The processes of atomic motion and changes in their magnetic moments were modeled in the LAMMPS software package [30]. This software package was created by a team of authors from Sandia National Laboratories and is distributed under the GPL license (i.e., it is freely available in the form of source codes). The additional package LAMMPS SPIN allows to perform numerical studies of magnetic systems and calculate the spin dynamics of atoms [22,31].

## Results and Discussion

The present paper deals with the solution of two problems related to the modeling of magnetic properties of cobalt nanostructures. The first problem was focused on confirming the adequacy of the used mathematical model and checking the convergence of the obtained numerical solutions. The second task investigated the self-organization of atomic spins in cobalt thin films and analyzed the dependence of nanofilm magnetic properties on their thickness. In both problems, there was no external magnetic field in the system, and the material structure corresponded to a face-centered cubic crystal lattice (fcc).

**Figure 1:**
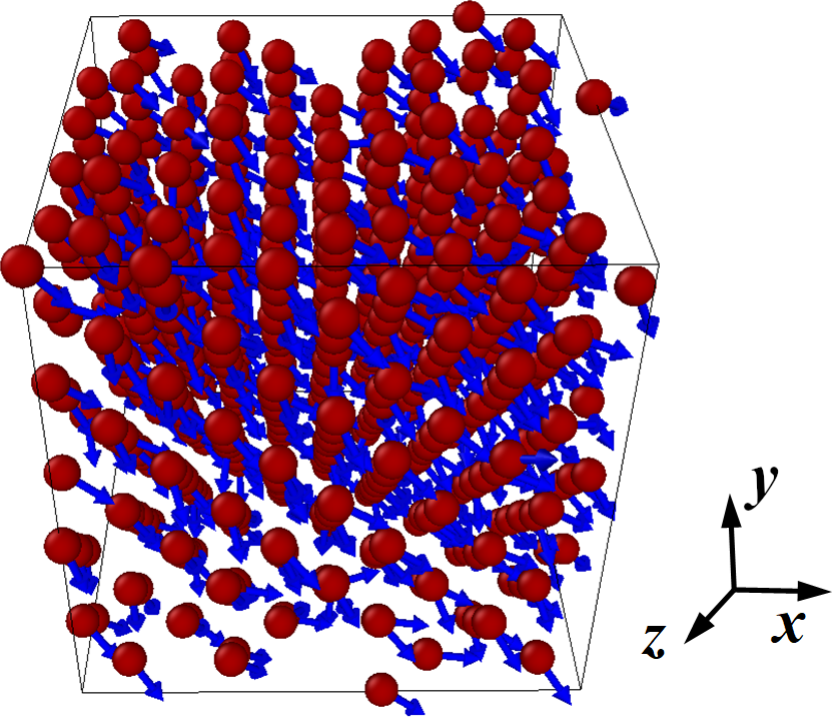
Appearance of a cobalt fcc crystallite (500 atoms) together with the orientation of atomic spins at the final relaxation stage for thermodynamic and spin temperatures of 300 K.

The size of the system in the first problem was small at 500 atoms (5 × 5 × 5 elementary crystal cells) and was due to the study of a similar system in [22]. The appearance of the modeled cobalt crystallite and the magnetic moments of its atoms are shown in Figure 1. Periodic boundary conditions were applied to the computational cell along all coordinate directions. The system was symmetric along all coordinate axes. The magnetic parameters of the exchange integral (Equation 7) were also chosen according to [22]. The magnetic behavior of the cobalt crystallite was considered in two stages. At the first stage (50 ps), relaxation of the system was performed, which resulted in mutual ordering of the spins of the atoms and orientation of the magnetization vector of the whole crystallite in some specific direction. At the second stage (10 ps), fluctuations of the magnetic values of the system stabilized after the relaxation stage were analyzed.

**Figure 2:**
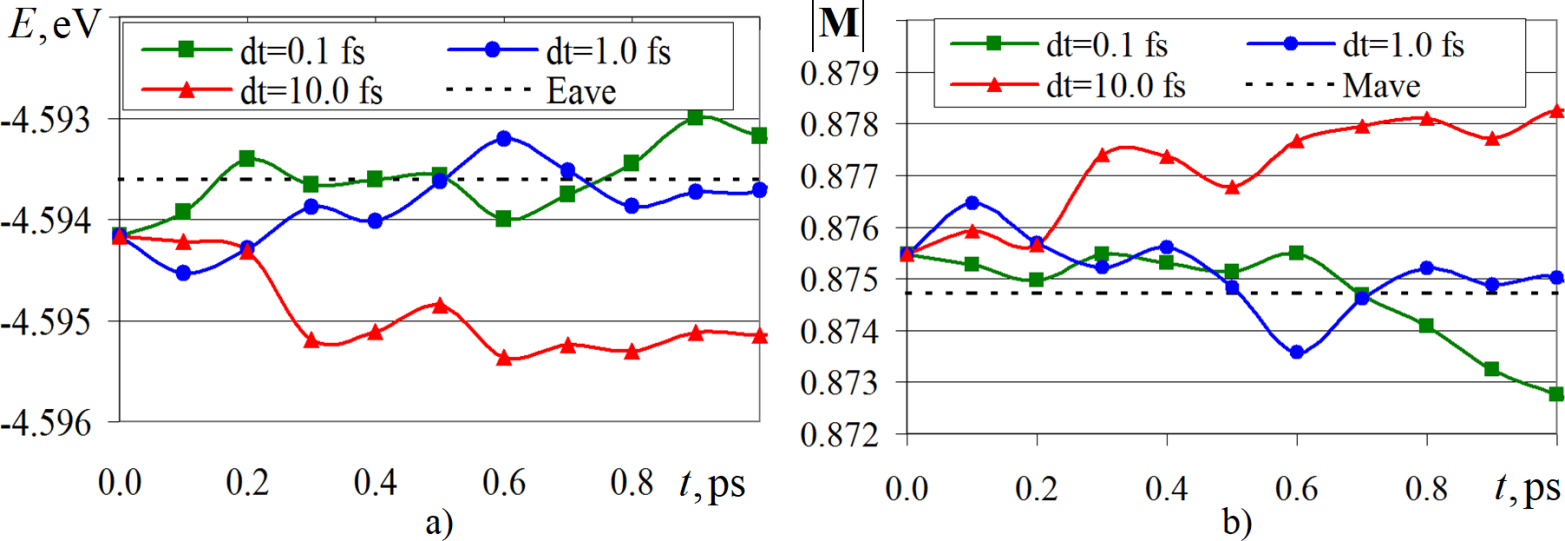
Variation of magnetic energy (a) and magnetization modulus (b) of a cobalt fcc crystallite (500 atoms) for different steps of integrating the systems of Equation 1 and Equation 2.

For the stage of fluctuations of magnetic quantities, the graphs of changes in magnetic energy and reduced magnetization modulus (takes values from 0 to 1) of a cobalt crystallite for integration steps d*t* = 0.1–10.0 fs were plotted. These graphs are selectively illustrated in Figure 2. For the other integration steps, the dependences have similar behavior. The dotted line in Figure 2 shows the average values of energy and magnetization modulus for an integration step of 0.1 fs. For simplicity in comparing the results with previously known data of other authors, the magnetic energy and magnetization modulus analyzed in this work were normalized with respect to the total number of atoms in the system.

Analysis of the plots in Figure 2 shows that small integration steps are characterized by smaller fluctuations of magnetic energy and magnetization modulus. The steps d*t* = 0.1 fs and d*t* = 1.0 fs lead to changes in the instantaneous values of the magnetic parameters near the average values of *E* = −4.5936 eV and |**M**| = 0.8747, respectively. Comparison of the average normalized magnetic energy and normalized magnetization modulus of a similar cobalt crystallite system from [22] gives values of *E* = −4.4900 eV and |**M**| = 0.9019, which correspond to a relative error value of 2% for energy and 3% for the magnetization modulus. The level of deviation of the parameters may be related to the fact that in [22] the modeling was carried out within the framework of the microcanonical ensemble, while in the present work a stochastic approach based on the Landau–Lifshitz–Hilbert equation was used.

For further analysis of the convergence of the numerical solutions and the influence of the time step on the fluctuations of the magnetic properties of the system, the relative deviations of the normalized magnetic energy Δ*E*(Δ*t*) and magnetization modulus Δ*M*(Δ*t*) were calculated. At the same time, additional averaging over the already performed time steps was performed for the considered quantities:


[8] $$\Delta E\left(\Delta t\right)=\frac{1}{N_{\text{step}}}\sum_{k=1}^{N_{\text{step}}}\left|\frac{E_{k}\left(\Delta t\right)-\langle E\rangle \left(\Delta t\right)}{\langle E\rangle \left(\Delta t\right)}\right|,$$

[9] $$\Delta M\left(\Delta t\right)=\frac{1}{N_{\text{step}}}\sum_{k=1}^{N_{\text{step}}}\left|\frac{M_{k}\left(\Delta t\right)-\langle M\rangle \left(\Delta t\right)}{\langle M\rangle \left(\Delta t\right)}\right|,\;M=\left|M\right|,$$

where *N*_step_ is the previously performed number of time steps; *E_k_*(Δ*t*) and *M_k_*(Δ*t*) are magnetic energy and magnetization modulus at the current time step; ⟨*E*⟩(Δ*t*) and ⟨*M*⟩(Δ*t*) are average values of the considered parameters over the entire time period.

**Figure 3:**
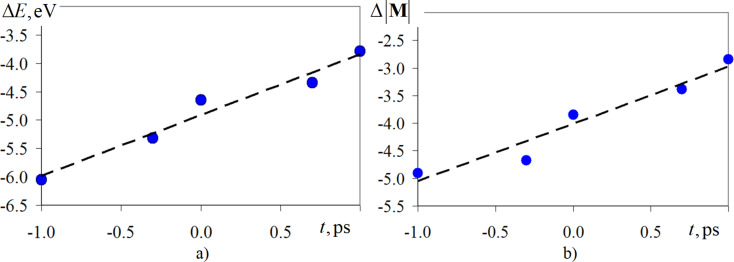
Dependences of the deviation of the relative normalized magnetic energy (a) and magnetization modulus (b) from different time steps of integration. The scales on the abscissa and ordinate axes are presented in decimal logarithm values.

The deviations of the magnetic parameters from Equation 8 and Equation 9 as a function of different integration steps are shown in Figure 3. For convenience in analyzing the data in Figure 3, the values along the abscissa and ordinate axes are given in logarithmic scale. The black dashed lines represent linear approximation functions, since the calculated points of the graphs are nearly a straight line. The approximation value for the deviation of magnetic energy Δ*E*(Δ*t*) was *R*^2^ = 0.96, for the deviation of magnetization modulus Δ*M*(Δ*t*) was *R*^2^ = 0.93.

A comparison of Δ*E*(Δ*t*) and Δ*M*(Δ*t*), obtained from the simulations in this work and the values from [22] indicates a satisfactory qualitative and quantitative agreement of the values. This analysis confirms that the modeling of magnetic properties and behavior of nanomaterials at the atomistic level should be carried out at integration time steps of no more than 1.0 fs. Typical spin dynamics models use a time step 0.1 fs, but the algorithm remains sufficiently accurate up to a time step of 10.0 fs. Therefore, for conducting computational experiments, an integration step of up to 10.0 fs can be used. According to [22], numerical results obtained with a time step of 0.1 fs show the greatest stability and robustness, since the characteristic time scales of changes in the magnetic moments of atoms are much smaller compared to their spatial counterparts. In the second problem, a unified integration step of 0.1 fs was used for all computational experiments.

In the second problem, crystalline cobalt (fcc) nanofilms containing 20 elementary crystal cells along the *x* and *y* axes were considered. The thickness of the films in the *z*-axis direction was varied in the range from 5 to 20 in steps of one elementary crystal cell. As studies show, the magnetic ordering in cobalt nanofilms with a thickness of less than 1.8 nm becomes unstable, and in the monolayer limit, it can be completely destroyed. When the film thickness is less than 1–2 monolayers (≈0.2–0.4 nm), the nanofilm ceases to be continuous, forming islands. This leads to random fluctuations in magnetization. Additionally, in monolayers and sub-monolayers, the number of nearest neighbors for cobalt atoms sharply decreases. This weakens the exchange interaction that stabilizes the ferromagnetic order. Disruption of lattice periodicity and a high density of defects exacerbate this problem. For these reasons, such studies are not included in this article. Thus, 16 computational experiments were realized in the second task, in which the thickness of the nanofilms was gradually increased and the investigated systems contained from 8,800 to 32,800 cobalt atoms. Along the horizontal axes (*x* and *y*), periodic boundary conditions were applied along the edges of the nanofilm, while vertically (*z* coordinate axis) the boundaries of the computational cell remained free.

The numerical realization of each individual computational experiment included two stages. At the first stage, the system proceeded through the stage of partial ordering of magnetic moments from the initial random distribution of spins and subsequent relaxation within 50 ps. This stage was necessary to exclude the influence of initial conditions on the magnetic characteristics further investigated. In the second stage, the magnetic properties of the previously energetically equilibrated cobalt nanofilm were investigated. The duration of the second stage was 10 ps. In both stages, there were no external influences on the system. The thermodynamic and spin temperatures were maintained at 300 K, with temperature damping parameters of τ*_t_* = τ*_s_* = 0.01 ps.

**Figure 4:**
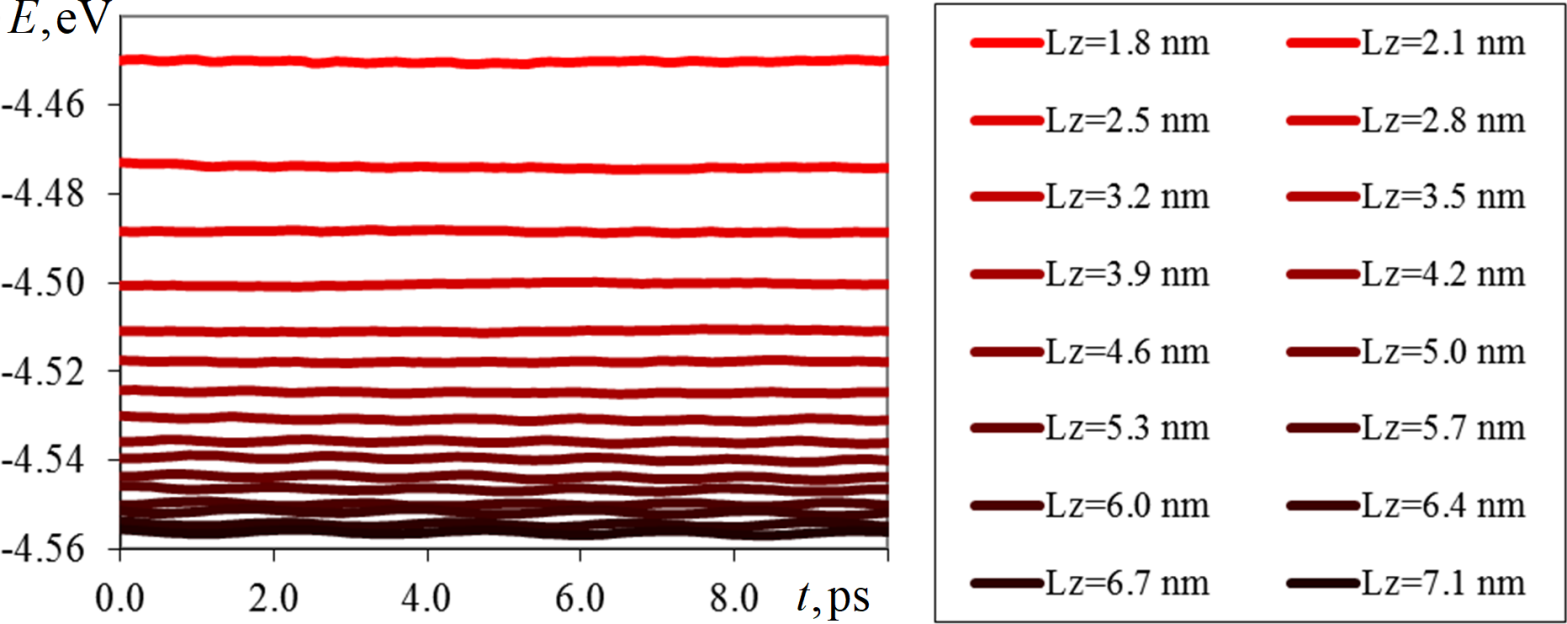
Variation of the magnetic energy of cobalt (fcc) normalized by the number of atoms in the system for nanofilms with thicknesses of 1.8–7.1 nm and thermodynamic and spin temperatures of 300 K.

**Figure 5:**
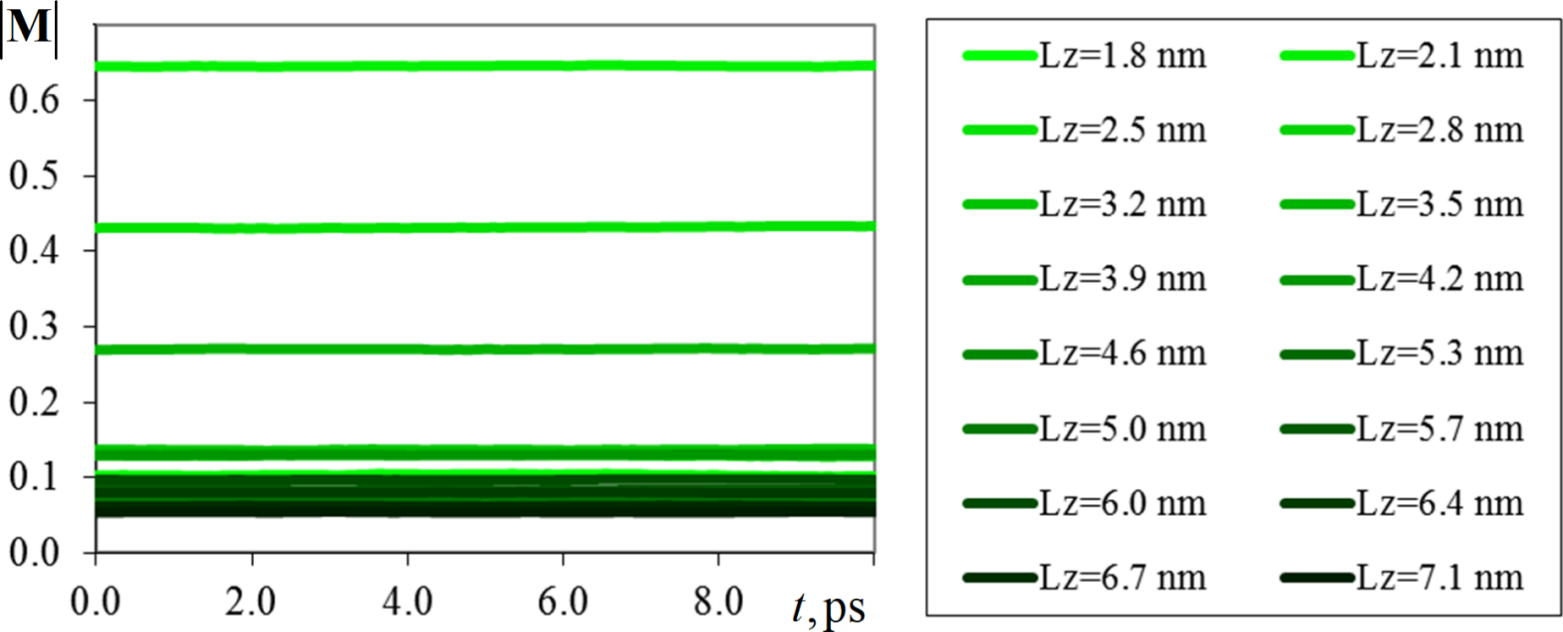
Variation of the magnetization modulus of cobalt (fcc) normalized by the number of atoms in the system, for nanofilms of thicknesses 1.8–7.1 nm and thermodynamic and spin temperatures of 300 K.

Magnetic energy and reduced magnetization modulus were considered as the investigated properties of cobalt nanofilms. As in the first problem, these quantities were normalized compared to the total number of atoms of the system for convenience of comparison. Figure 4 and Figure 5 are plots of the time variation of magnetic energy and magnetization modulus for the whole series of computational experiments. For clarity, the shade of the lines in the graphs is depicted in a darker color with increasing film thickness *L_z_*, where *L_z_* = 1.8 nm corresponds to five elementary crystal cells, and *L_z_* = 7.1 nm to 20 elementary crystal cells.

As can be seen from the graphs in Figure 4 and Figure 5, the magnetic energy of the systems at this stage of the computational experiment stabilizes and does not significantly change over time; only its insignificant fluctuations are noticeable. Increasing the thickness of the nanofilm, in turn, leads to a decrease in the value of magnetic energy, in connection with which a gradient change of colors in the graphs in Figure 4 is observed. The most significant differences in the magnetic energy values were obtained for thin films *L_z_* = 1.8–3.2 nm. For thicker cobalt nanofilms, the values of normalized magnetic energy are in the range of −4.56 to −4.53 eV. Such behavior of the magnetic energy can be related to the fact that with increasing thickness of the nanofilm, the fraction of its surface atoms decreases and the influence of various surface effects decreases. As the number of crystalline layers of the nanofilm increases, the modeled system approaches a bulk material in terms of its physical properties. That is why, from the point of view of functional characteristics, thin-film ferromagnetic nanostructures are of the greatest interest.

**Figure 6:**
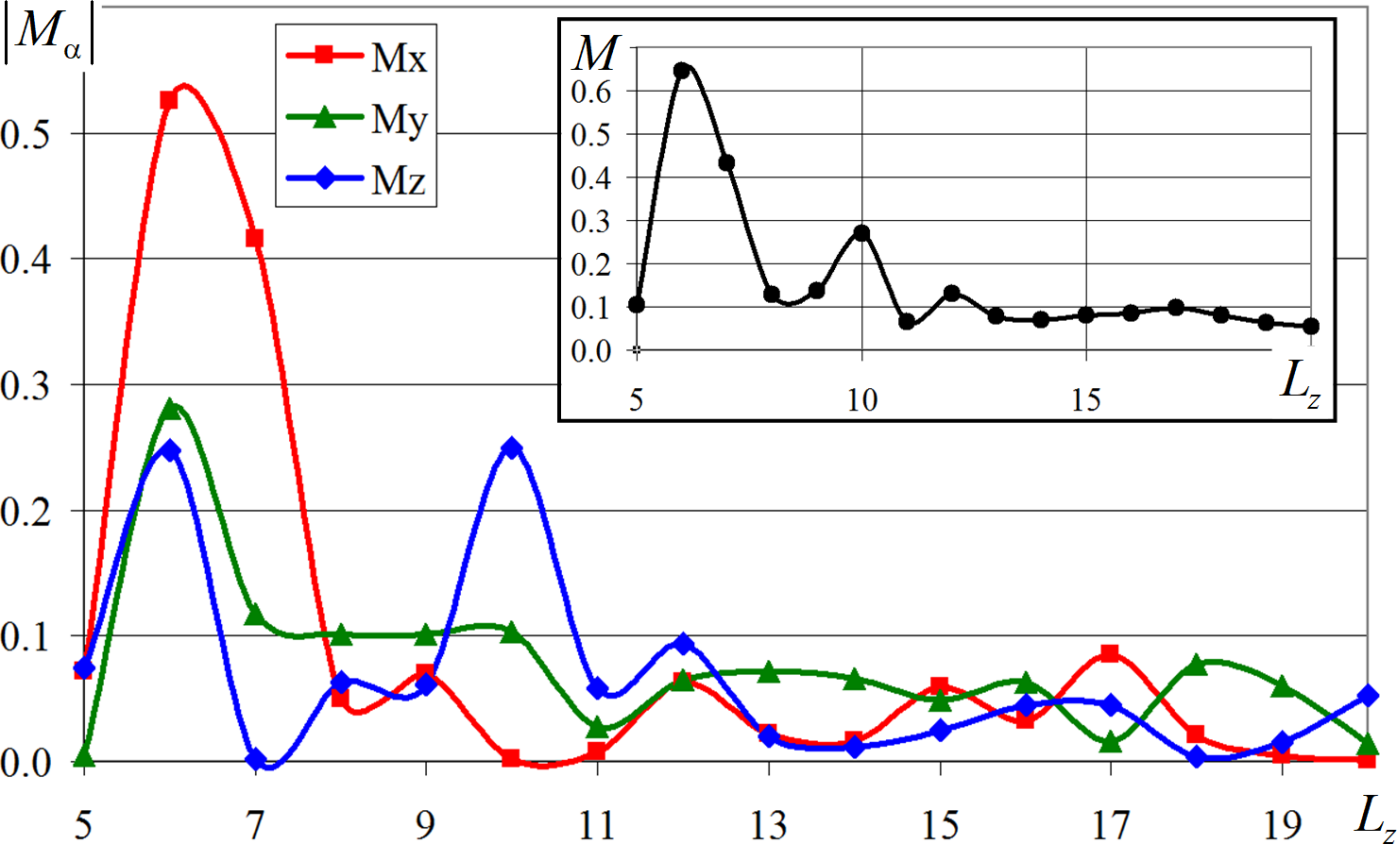
Variation of magnetization vector components (in absolute values) *M*_α_,α = {*x*,*y*,*z*} as a function of cobalt nanofilm thickness measured in crystalline layers *L_z_*, *M* = |**M**|.

Analysis of the graphs in Figure 5 shows that for the whole series of computational experiments after the relaxation stage, the magnetization modulus value also slightly changes in time. The simulation results indicate that as the thickness of the nanofilm increases, there is a tendency to decrease the value of its magnetization modulus. Most of the values are concentrated in the range from 0.05 to 0.13. However, there are a number of exceptions to the smooth gradient variation of the magnetization modulus in Figure 5, indicating its nonlinear dependence on the thickness of the investigated nanofilm. Since on the basis of Figure 5 it is difficult to speak about the type of the obtained dependence, we have plotted graphs of changes in the individual components of the magnetization vector (in absolute values), which are presented in Figure 6. In a separate inset in Figure 6, the behavior of the magnetization modulus for cobalt nanofilms of different thicknesses is shown. As *M*_α_ in Figure 6, the time-averaged value of the components of the magnetization vector was used.

The study of the dependences plotted in Figure 6 indicates that the film thickness has a significant effect on the magnetization value. A three-modal distribution of the magnetization vector components with peaks is observed at nanofilm thicknesses of 6, 10, and 12 crystal cells. The intensity of the peaks decreases with increasing thickness of the cobalt layers, which is clearly visible in Figure 6. The value of each next mode decreased in absolute value by more than 50% compared to the previous one. It is also interesting to notice that all three modal values were obtained for nanofilms with an even value of crystalline layers. The influence of the nanofilm thickness on the magnetization value significantly decreases for film thicknesses above 4.5 nm. In this range of values, local oscillations of the magnetization vector components occur, but they do not significantly affect the final modulus value.

**Figure 7:**
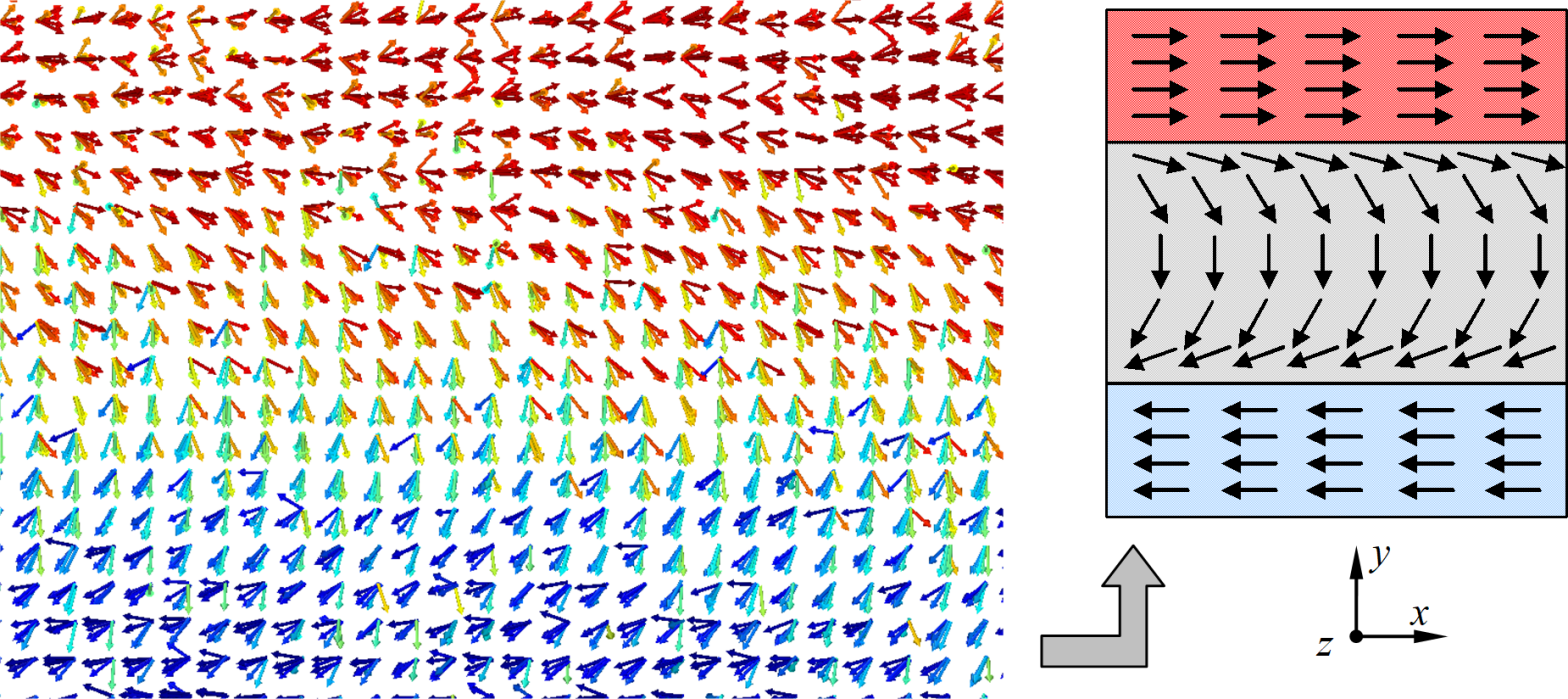
Formation of Neel domain walls in a cobalt nanofilm after the relaxation stage.

The presence of the obtained peaks of the magnetization vector components distribution in Figure 6 may be due to surface effects in thin films and the formation of domain walls. Depending on the symmetry of the modeled crystalline structures, the joint orientation of magnetic moments of atoms and their way of turning on the boundary, the types of domain walls may differ. For example, Bloch-type domain walls, Neel-type domain walls, walls with reduced angle, and cylindrical domain walls are known. Analysis of magnetic moments of atoms mutually ordered as a result of modeling has shown that, for some cases of calculations for thin films of cobalt, the formation of Neel domain walls is typical [32–34]. An illustration of the resulting Neel domain wall is given in Figure 7, which shows the orientation of atomic spins obtained by numerical simulation after the relaxation and equilibration of the system.

As can be seen in Figure 7, the reversal of the magnetic moments of individual atoms occurs in the plane of the nanofilm. After the reversal, the spins of the atoms are oriented in the opposite direction, which is demonstrated in the right part of Figure 7. For nanofilms with a thickness of 13–20 crystalline layers, the formation of Neel walls dividing the modeled sample into domains with opposite directions of magnetic moments is observed. As a result, the total magnetization of the nanofilm is formed in the range of 0.07–0.01. It is known that the Neel domain walls occur in thin films with a thickness of 100 nm or less, which agrees well with the simulation results obtained. Since it is typical for ferromagnetic materials to form domains and domain walls due to the strong interaction of spin moments of nearby atoms, the data of computational experiments confirm the ferromagnetic nature of the magnetic behavior of cobalt nanofilms.

**Figure 8:**
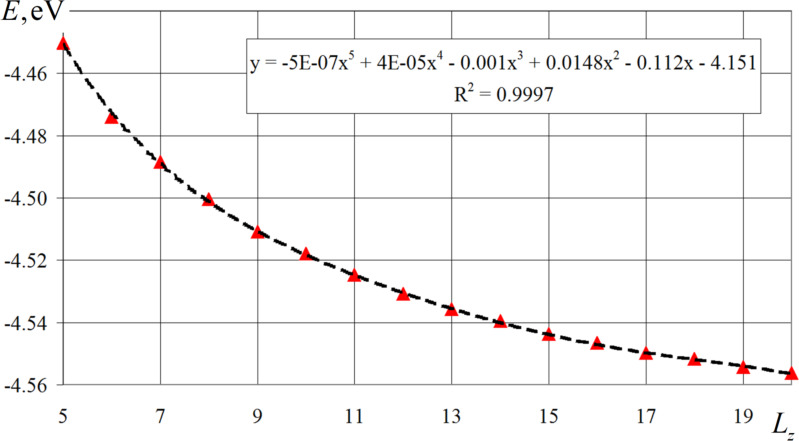
Variation of cobalt magnetic energy as a function of nanofilm thickness measured in crystalline layers.

For nanofilms with thicknesses of 6, 10, and 12 crystalline cells, which corresponded to the peaks and maximum values of the magnetization vector, the formation of domains of approximately the same size with antiparallel spin distribution was not observed. The predominant orientation of magnetic moments of cobalt atoms in a certain direction led to bursts and jumps in the values of individual components (*x*, *y*, *z*) of the magnetization vector and its modulus as a whole, which is clearly visible in the graphs in Figure 6. Due to the fact that it is difficult to assess the peculiarities of the connection between the magnetic energy of the modeled nanofilms and their thickness in Figure 4, the graph for these values was separately plotted. Figure 8 shows a nonlinear dependence of the magnetic energy on the thickness of the modeled nanofilm. The given values of magnetic energy were pre-averaged over time for a period of 10 ps after the relaxation step. The smooth change of the graph in Figure 8 may be due to a gradual and uniform decrease in the fraction of surface atoms of the nanofilms as their thicknesses increase.

The graph of calculated magnetic energy values is well approximated by a polynomial dependence of the fifth degree (the equation is shown in the frame in Figure 8). The value of reliability of the constructed trend line is 0.9997. Simplification of the approximating function to a polynomial of the third degree (the coefficients in front of the 4th and 5th order terms are quite small) also brings about good results of the trend line at the reliability level of 0.9978. Linear approximation gives a lower indicator of the reliability level (i.e., 0.8848).

## Conclusion

The mathematical model considered in this work allows us to investigate the magnetic behavior of a nanoscale system taking into account spin and interatomic interactions. The mathematical model is based on the joint solution of the Langevin and Landau–Lifshitz–Hilbert equations. In this work, the multi-particle potential of the modified immersed atom method, which is well established for solving such problems, is used to describe the interatomic interaction.

Numerical studies of the magnetic behavior of cobalt-based nanofilms were carried out in two stages. Analysis of the convergence of numerical solutions and evaluation of the time step effect on the fluctuations of the magnetic properties of the system showed that modeling of magnetic properties and behavior of nanomaterials at the atomistic level should be carried out at integration time steps of no more than 1.0 fs. A time step of 0.1 fs was used for the main series of computational experiments.

The study of the influence of the cobalt nanofilm thickness on the magnetic behavior of the system showed a decrease in the magnetic energy with increasing film thickness. It should be noted that the magnetic energy nonlinearly changes with increasing thickness of the cobalt nanofilm. The magnetization rate of the modeled systems also does not significantly change with time. The analyzed dependence of the magnetization norm on the thickness of the studied nanofilms illustrate the nonlinear change of this magnetic characteristic with peaks at nanofilm thicknesses of 6, 10, and 12 crystal cells. The effect of the nanofilm thickness on the magnetization value is leveled off at nm. The effects observed in the investigated films can be due to surface effects in thin films and the formation of Neel domain walls.

The dependence of the magnetic energy on the thickness of the modeled nanofilm has a nonlinear form and is well approximated by a polynomial of degree 5. Due to insignificant coefficients in front of the 4th and 5th order terms, the approximation function can be simplified to a 3rd order polynomial without significant decrease in the accuracy level. The gradual nonjump-like change of magnetic energy with increasing thickness of the studied cobalt nanofilms can be associated with a uniform and proportional decrease in the fraction of surface atoms in the thickened films.

## Data Availability

Data generated and analyzed during this study is available from the corresponding author upon reasonable request.
